# A Combined Effect of Self and Reward: Relationship of Self- and Reward-Bias on Associative Learning

**DOI:** 10.3389/fpsyg.2021.647443

**Published:** 2021-06-17

**Authors:** Lingyun Wang, Yuxin Qi, Lihong Li, Fanli Jia

**Affiliations:** ^1^School of Psychology, Northeast Normal University, Changchun, China; ^2^Department of Psychology, Seton Hall University, South Orange, NJ, United States

**Keywords:** associative learning, self-bias, reward-bias, significant others, perceptual matching

## Abstract

Previous studies have demonstrated that individuals process information related to themselves or a high reward quickly and have referred to this as self-bias or reward-bias. However, no previous study has presented self- and reward-bias simultaneously. The present study investigated perceptual processing using the associated learning paradigm when both self and reward were prioritized (condition of double salience) as well as when only self or reward was prioritized (condition of single salience). The present study established these two conditions by manipulating self-relevance (self vs. stranger in Experiment 1; self vs. friend in Experiment 2). The results showed that (1) when the self was pitted against a stranger and received a high or low reward, perceptual processing of the participants mainly involved self-bias (Experiment 1); (2) when the self was pitted against a friend, perceptual processing involved both self-bias and reward-bias (Experiment 2). The study revealed a complex relationship between self- and reward-bias, which depends on the degree of affinity between oneself and others.

## Introduction

Self-relevance and monetary rewards are two important social factors that promote the cognitive processing and behavior of an individual. On the one hand, self-related information can improve cognitive processing (perception, attention, and memory) more effectively than other-related information (Sui and Humphreys, 2015c). Humphreys and Sui (2016) discussed the relationship between self and attention and found that the self-related name and self-related face as targets required very few resources for processing. They argued that self-related processing guided attention distribution in a relatively automated manner (e.g., self-attention network (SAN)). For example, in a shape–label perceptual matching task (Sui et al., 2012), the shape associated with a self-label was matched more quickly and accurately than when the shape was associated with an other-label. Furthermore, in a spatial working memory task, self-related information was prioritized and maintained, showing self-superiority in working memory (Yin et al., 2019).

On the other hand, rewards can also influence cognitive processing and improve attentional selection (Pessoa and Engelmann, 2010; Chelazzi et al., 2013; Anderson, 2016, 2019; Miendlarzewska et al., 2016; Failing and Theeuwes, 2018). Chelazzi et al. (2013) categorized the influence of reward on selective attention into two forms. One form is incentive-motived learning, where rewards are used as incentive motivation to actively prioritize the attention of an individual to specific stimuli or positions that give rewards. The second form is reward-mediated attentional learning, where reward is the feedback given to the correct response to a specific stimulus. This approach allows participants to learn the connection between stimulus and reward, which directly increases their priority attention to the stimulus. For example, a stimulus associated with a reward in a previous stage would automatically capture the attention of an individual when presented as a task-independent distraction in the next stage, increasing target search time (Anderson et al., 2011; Anderson, 2016, 2019).

Recent studies have supported both voluntary and nonvoluntary influences of reward on perception and attention. Failing and Theeuwes (2018) reviewed recent studies and pointed out that the mechanism that rewards affect selective attention is not the result of top-down or bottom-up processing but the result of the delayed effect of attention input “history.” In other words, reward-induced attention input can continuously drive selective visual attention in various tasks and methods (e.g., Stroop task, spatial curing task, working memory task, visual searching task, and neuroimaging). They concluded that reward stimuli increase the voluntary priority of attention by motivating the cognitive processing voluntarily. In addition, Anderson et al. (2011) demonstrated that reward-related stimuli capture attention by associating non-salient, task-irrelevant stimuli with reward. In the visual search task (e.g., searching for green and red items), researchers tested the reward magnitude (e.g., high and low reward) on the performance of the participants. They found that the stimulus previously associated with high reward involuntarily captured attention in a subsequent visual search task. However, an empirical research that investigates both self-relevance and reward is limited. Therefore, the present research aims to clarify how self-relevance and monetary rewards might jointly influence behaviors and cognitive processes.

Northoff and Hayes (2011) were pioneers in conceptualizing a relationship between self-relevance and reward based on a neural mechanism research. They proposed three models to explain the relationship between self and reward: the Integration Model [“self-specific processing and reward-related processing are nearly identical”]; the Segregation Model [“self-specific processing and reward-related processing are nearly unrelated”], and the Parallel Processing Model [“the self is processed in parallel with lower-order reward processes”] (p. 2). To verify the relationship models for self-relevance and monetary rewards, Sui, Yankouskaya, and other researchers used several behavioral studies to find whether self-relevance and monetary rewards have similar processes. These studies all used the associative matching paradigm, which allowed participants to associate several unbiased geometric shapes with self-relevance (such as the self, a friend, or a stranger) or a monetary reward (such as a high, medium, or low amount). To establish the relationship, the researchers asked the participants to judge the matching between shapes (e.g., a triangle) and labels. The results showed that the reaction time (RT) when judging matches with self-relevance or high monetary rewards was faster than when judging matches with other-relevance or low monetary rewards (Sui et al., 2012). The results suggest the emergence of a self-bias (information with high self-relevance is processed faster than information with low self-relevance, such as information related to friends or strangers) and a reward-bias (high monetary rewards are processed faster than low monetary rewards).

In contrast, studies using the redundancy gains paradigm have supported the Segregation Model. Redundancy gain is the increase in performance when participants are presented with two identical targets (e.g., a combined effect) compared to when they are presented with a single target (e.g., a single effect; Miller, 1982). Sui and Humphreys (2015a) used this paradigm to test the combined effects of self-bias and reward-bias on stimulus integration. In their experiments, participants were asked to associate two shapes with self (e.g., circle and hexagon – you) and another two shapes with their best friend (square and rectangle – friend) and then a single shape, where two identical or nonidentical shapes were presented to be identified (Experiments 1 and 2). To compute the redundancy gains, researchers used the RTs of “1-item trials minus the RTs on 2-item trials” (Sui and Humphreys, 2015a). They found that the redundancy gain of self-bias exists at both the perceptual and the conceptual levels. However, no significant difference in redundancy gain was found between high-reward and low-reward associations (Sui and Humphreys, 2015a,b).

A recent study conducted by Yankouskaya et al. (2020) using the drift-diffusion approach supports the Parallel Processing Model. The drift-diffusion approach tests the effects of self-bias and reward-bias by “de-componenting” different stages of perceptual decision-making. Specifically, researchers can estimate a “rate of information accumulation” (e.g., correct and incorrect responses accumulating over time) to enable comparisons between different cognitive processes (Voss et al., 2004). In a recent experiment using the drift-diffusion approach, Yankouskaya et al. (2020) found that the accuracy and RTs of the participants were consistently affected by self-bias and reward-bias, which suggests a common effect of personal relevance and reward values on perceptual decisions. In addition, they did not find a qualitative difference between the two dimensions of behavioral performance (Yankouskaya et al., 2020), which supports the Parallel Processing Model.

In summary, when self-relevance and rewards are processed independently, results on behaviors indicate self-bias and reward-bias. However, there are certain differences in the mental representation and neural representation of the two processes. These differences may be due to the different sources of self-bias and reward-bias. Self-bias originates from within the individual and is related to the internal motivation of the individual. The process is relatively automatic (Sui et al., 2014; Yin et al., 2019). In contrast, reward-bias is mostly derived from the external motivation of an individual, and it may be related to the expectations of reward outcomes and implicitly learned associations [see example of the publications from the laboratories held by Anderson et al. (2011)]. In other words, self-relevance and monetary rewards promote individual behavior and cognitive processing, both internally and externally. Although self-relevance and monetary rewards are independent of each other, they are also carried out simultaneously in daily life. However, no previous research has examined how self-relevance and rewards influence cognitive processes simultaneously.

In the present study, we adopted an associative matching paradigm (Sui et al., 2012), where participants learned mental associations between geometric shapes (e.g., circles, triangles, squares, or hexagons) and labels (self and reward) and then judged several shape–label pairings. Rather than separating the social label from the reward label as in the previous studies, the current experiments were designed to study them simultaneously. Thus, two conditions were offered, namely, double salience (self-relevance and a monetary reward are both prioritized) and single salience (either self-relevance or a monetary reward is prioritized). Under the condition of double salience, shape 1 represented self-high monetary reward, and shape 2 represented other-low monetary reward. Under the condition of single salience, shape 1 represented self-low monetary reward (with high self-relevance), and shape 2 represented other-high monetary reward (with high reward). According to previous studies, individuals prioritize the self and rewards to the same degree. Therefore, in the matching task, processing differences between the two shape–label pairs resulted from the combined effect of self-prioritization (differences between high and low self-relevance) and reward-prioritization (differences between high and low monetary rewards).

Under the condition of double salience, both self and reward were prioritized. We expected that there would be significant differences for accuracy and RT on matching judgments between self-high reward shapes and other-low reward shapes. Under the condition of single salience, either self or reward was prioritized. If participants processed the self-low reward shapes faster than other-high reward shapes, it would indicate that self-relevance is more important than reward. If participants processed the other-high reward shapes faster than the self-low reward shapes, it would indicate that the reward is more important than self-relevance. If participants showed no significant difference in the processing of self-low reward shapes and other-high reward shapes, it would imply that self-relevance and rewards are equivalent and act simultaneously.

In addition, previous studies have shown that the degree of affinity between the self and others affects the magnitude of self-bias and sensitivity to rewards (Sui and Humphreys, 2015d). Therefore, the present research also explored the influence of the type of other person on the interaction of self-relevance and monetary rewards when the other person was a stranger (Experiment 1) and when the other person was a friend (Experiment 2). Based on the results of the previous research results, we hypothesized that, when self-relevance and a monetary reward act simultaneously, the role of self-prioritization would be greater than that of reward-prioritization. We also expected that reward-prioritization should be higher when self-relevance is low (e.g., friend).

## Experiment 1

### Method

#### Participants

*A priori* power analysis was conducted using G∗power 3.1.9.2 (Faul et al., 2007) to test the difference between two shapes using a two-tailed test, alpha of 0.05, and a medium effect size (*d* = 0.5). The result indicated that a total sample of 34 participants was required to achieve a power of 0.80.

Thirty-three participants were randomly selected [12 males and 21 females, 18–27 years old, with an average age of 21.39 (*SD* = 2.70 years)]. One participant was removed from the analysis as the learning stage was not completed. All the participants were college students with normal or corrected vision. They participated voluntarily and had not participated in similar experiments before. Each participant was given monetary compensation ($15) after the completion of the experiment. This experimental protocol was approved by the Ethics Committee of the local University. The authors agree to share data and materials upon request.

#### Materials

The stimulus was presented on a iiyama 22-inch CRT monitor (screen resolution of 1,024 × 768, 85 Hz), with eyes 60 cm away from the screen. E-Prime 2.0 was used for programming and data collection. The stimulus had a gray background (RGB values: 128, 128, 128). The experiment adopted a variant of the associative matching paradigm (Sui et al., 2012). First, participants were asked to associate a geometric shape with two social identity labels, namely, self-relevance (self or stranger) and a monetary reward (high or low value). Then, the participants were asked to perform the matching judgment of shapes and labels (a social label and a monetary label). Geometric shapes (circles, triangles, squares, or hexagons, viewing angle 4.7° × 4.7°) appeared above the central white fixation point “+” (0.8° × 0.8°), at an angle of 3.67° from the central fixation point. Two labels (oneself, a stranger, $50, or $200, 2.4°~3.6° × 1.2°) were displayed horizontally (the horizontal distance between the centers of the two labels was 8.15°) below the fixation point, at an angle of 2.44° from the central fixation point. The associations of the shapes and labels were counterbalanced across participants.

#### Design

The experiment had a within-subject design with 2 (salience: double salience/single salience) × 2 (shapes: shape 1/shape 2) × 3 (matching condition: double match/single match/non-match). Salience is a between-block variable (after counterbalancing), whereas shapes and matching conditions are both in-block variables. Double salience is the condition wherein self-related and reward-related information goes in the same direction. Under double salience, shapes 1 and 2 were, respectively, associated with self-high reward and stranger-low reward. Single salience is the condition wherein the two dimensions go in opposite directions. Under single salience, shape 1 was associated with self-low reward, and shape 2 was associated with stranger-high reward. Indexes of performance difference between critical pairs were calculated for the conditions of double and single salience separately to highlight eventual differences emerging as a function of the condition of salience. The dependent variables were accuracy and RT.

#### Procedure

**Figure 1:**
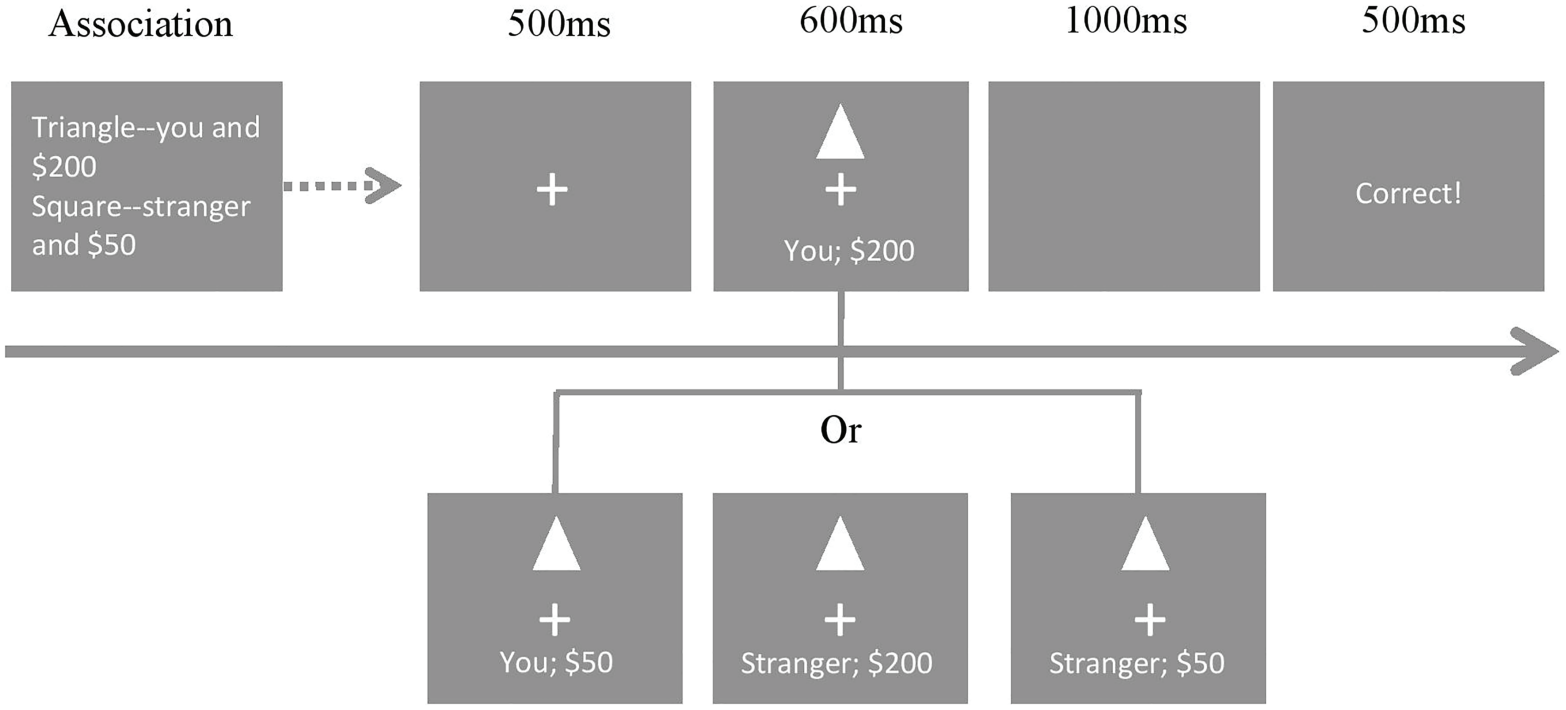
Experimental procedure. This procedure is an example of the condition of double salience. The triangle represents self-high reward shapes with double matching (“you and $200”), single matching (“you and $50” or “a stranger and $200), and non-matching (“a stranger and $50) combinations.

The experiment used a 2-stage associative matching paradigm. In the first associative learning stage, participants were asked to learn associations, such as “A triangle represents yourself and $200 (high value), and a square represents a stranger and $50 (low value).” Participants were asked to memorize these associations without a response or a time limit. In the matching stage, participants judged whether shape–label pairings matched (e.g., triangle-you, $200; triangle-you, $50; and triangle-stranger, $200) or mismatched (e.g., triangle-stranger, $50; see Figure 1). There were two phases within the matching stage. First, the central gaze point appeared in the center of the screen for 500 ms, and then, the shape–label pairing was presented for 600 ms. Participants were asked to judge whether the shape was correctly assigned to the person/reward by pressing the response buttons as quickly and accurately as possible. Next, a 1,000 ms blank screen was shown to ensure all the responses could be collected. Feedback was given for 500 ms. Participants were expected to press the “m” (or “n”) key when the shape matched both the social label and the reward label (double matching condition) and when only one label matched (single matching condition). They were asked to press the “n”(or “m”) key when the shape did not match the labels (non-matching condition). Participants were allowed to repeat practice trials before the matching task. Each salience condition (single and double) had six blocks. Each block had 120 trials that involved two shapes with three randomly presented matching conditions. After each block, the participants took a short break. A total of 1,440 trials were presented. To balance the amount of key pressing, the ratio of double matching, single matching, and non-matching condition trials was 1:2:3.

### Results

**Figure 2:**
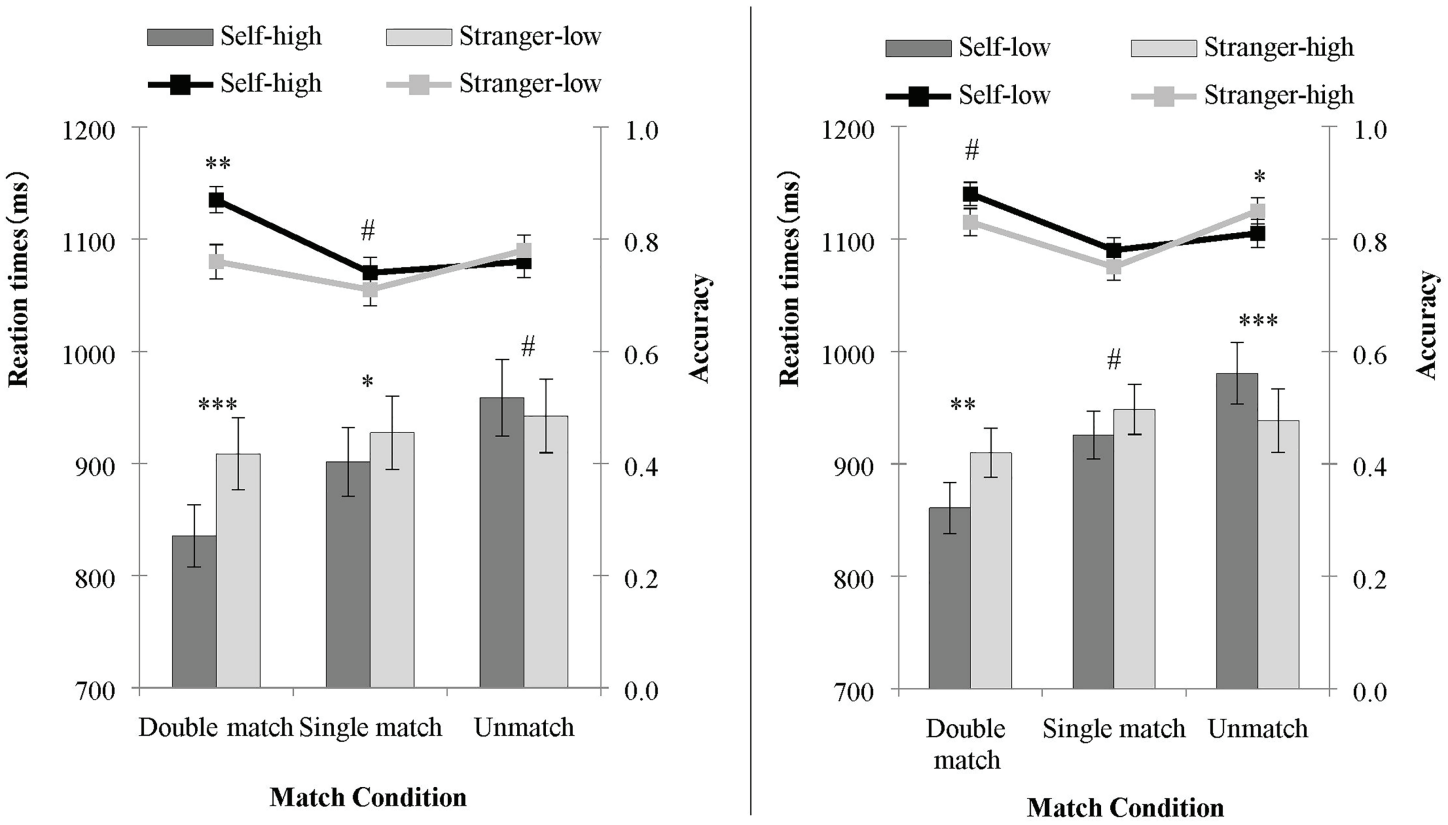
The accuracy and reaction time of judgments under the double salience condition (left) and single salience condition (right) in Experiment 1. The line represents accuracy, and the bars represent the RT. ^∗∗∗^*p* < 0.001; ^∗∗^*p* < 0.01; ^∗^*p* < 0.05; ^#^*p* = 0.1–0.05.

Outliers (RT less than 200 ms) were excluded, accounting for 2.69% of the total data. Because double salience and single salience were between-block designs, the data under the two conditions were analyzed separately. A repeated measures analysis of variance (rANOVA) of 2 (shapes: shape 1/shape 2) × 3 (matching condition: double match/single match/non-match) was conducted on the accuracy and RT of matching judgments. The result is shown in Figure 2.

#### Double Salience

##### Accuracy

The results showed a significant main effect of shape, *F* (1, 32) = 7.12, *p* = 0.01, $n_{p}^{2}$ = 0.19. The matching judgment of the self-high reward shapes was significantly more accurate than that of the stranger-low reward shapes. The main effect of the matching condition was also statistically significant, *F* (2, 64) = 4.50, *p* = 0.015, $n_{p}^{2}$ = 0.12. The accuracy of a double match was higher than the accuracy of a single match (*p* < 0.01). There was no significant difference between a single match and a non-match (*p* = 0.21). The interaction between the shape and the matching condition was significant, *F* (2, 64) = 7.83, *p* < 0.01, $n_{p}^{2}$ = 0.20. A paired sample *t*-test of the shapes was conducted for the three matching conditions. In the double matching condition, the accuracy rate of processing self-high reward shapes was higher than that of stranger-low reward shapes, *t* (32) = 3.24, *p* < 0.01, Cohen’s *d* = 0.56. For the single matching condition, the accuracy rate for processing self-high reward shapes was higher than that of stranger-low reward shapes, *t* (32) = 1.84, *p* = 0.076, Cohen’s *d* = 0.25. There was no significant difference in the processing accuracy of the two shapes in the non-matching condition, *t* (32) = 1.30, *p* = 0.20.

##### Reaction Time

Reaction times (RTs) for only the correct responses were analyzed. The main effect of shape was statistically significant, *F* (1, 32) = 9.10, *p* < 0.01, $n_{p}^{2}$ = 0.22. The RT on matching judgments of the self-high reward shapes was significantly faster than the stranger-low reward shapes. The main effect of the matching condition was statistically significant, *F* (2, 64) = 28.27, *p* < 0.001, $n_{p}^{2}$ = 0.47. Participants responded faster in the double matching condition than they did in the single matching condition (*p* < 0.001) and the non-matching condition (*p* < 0.001), with a faster RT in the single matching condition than in the non-matching condition (*p* < 0.01). The interaction between shape and matching condition was statistically significant, *F* (2, 64) = 19.76, *p* < 0.001, $n_{p}^{2}$ = 0.38. A paired sample *t*-test of shape was performed for the three matching conditions. In the double matching condition [*t* (32) = 4.38, *p* < 0.001, Cohen’s *d* = 0.76] and the single matching condition [*t* (32) = 2.47, *p* = 0.019, Cohen’s *d* = 0.43], the RT for processing self-high reward shapes was faster than that of stranger-low reward shapes. In the non-matching condition, the RT for processing self-high reward shapes was marginally slower than that of stranger-low reward shapes, *t* (32) = 2.00, *p* = 0.055, Cohen’s *d* = 0.36.

#### Single Salience

##### Accuracy

The main effect of the shape was not statistically significant, *F* (1, 32) = 1.01, *p* = 0.323, whereas the main effect of the matching condition was statistically significant, *F* (2, 64) = 8.52, *p* < 0.01, $n_{p}^{2}$ = 0.21. Response accuracy of the participants was higher in the double matching condition than in the single matching condition (*p* < 0.001). However, participants reported more accuracy in the non-matching condition than in the single matching condition (*p* = 0.010). There was no significant difference between the double matching and the non-matching condition (*p* = 0.237). More importantly, the interaction between the shape and the matching condition was significant, *F* (2, 64) = 6.01, *p* < 0.01, $n_{p}^{2}$ = 0.16. A paired sample *t*-test of shape was conducted for the three matching conditions, respectively. In the double matching condition, the accuracy rate for processing self-low reward shapes was higher than that of stranger-high reward shapes, *t* (32) = 2.21, *p* = 0.051, Cohen’s *d* = 0.41. For the single matching condition, there was no significant difference, *t* (32) = 1.40, *p* = 0.17. In the non-matching condition, stranger-high reward shapes were processed more accurately than self-low reward shapes, *t* (32) = 2.97, *p* < 0.01, Cohen’s *d* = 0.59.

##### Reaction Time

The main effect of shape was not significant, *F* (1, 32) = 0.95, *p* = 0.338. The main effect of the matching condition was significant, *F* (2, 64) = 16.46, *p* < 0.001, $n_{p}^{2}$ = 0.34.

The RT in the double matching condition was faster than in the single matching and non-matching conditions (*ps* < 0.001). There was no significant difference between the single matching and non-matching conditions (*p* = 0.112). Moreover, there was a significant interaction between the shape and the matching condition, *F* (2, 64) = 18.88, *p* < 0.001, $n_{p}^{2}$ = 0.37. A paired sample *t*-test was performed for the three matching conditions, respectively. In the double matching condition, the RT for processing self-low reward shapes was faster than that of stranger-high reward shapes, *t* (32) = 2.80, *p* < 0.01, Cohen’s *d* = 0.49. In the single matching condition, the RT for processing self-low reward shapes was marginally faster than that of stranger-high reward shapes, *t* (32) = 1.94, *p* = 0.062, Cohen’s *d* = 0.34. In the non-matching condition, the RT for processing self-low reward shapes was slower than that of stranger-high reward shapes, *t* (32) = 4.30, *p* < 0.001, Cohen’s *d* = 0.76.

#### Differences in Reward-Bias in Conditions of Double and Single Salience

Based on the results of previous studies (e.g., Sui et al., 2016) that the effects of self-bias and reward-bias in the condition of double salience are equally prioritized (self-high reward vs. other-low reward), it is not surprising that participants in the present study processed self-high reward shapes faster than stranger-low reward shapes. In the condition of single salience, where self-bias and reward-bias were reversed (self-low reward vs. other-high reward), the self-low reward shapes were processed better than the other-high reward shapes. The results might only indicate that the role of self-bias is greater than the role of reward-bias. However, these results could not reveal the contribution of reward-prioritization.

**Figure 3:**
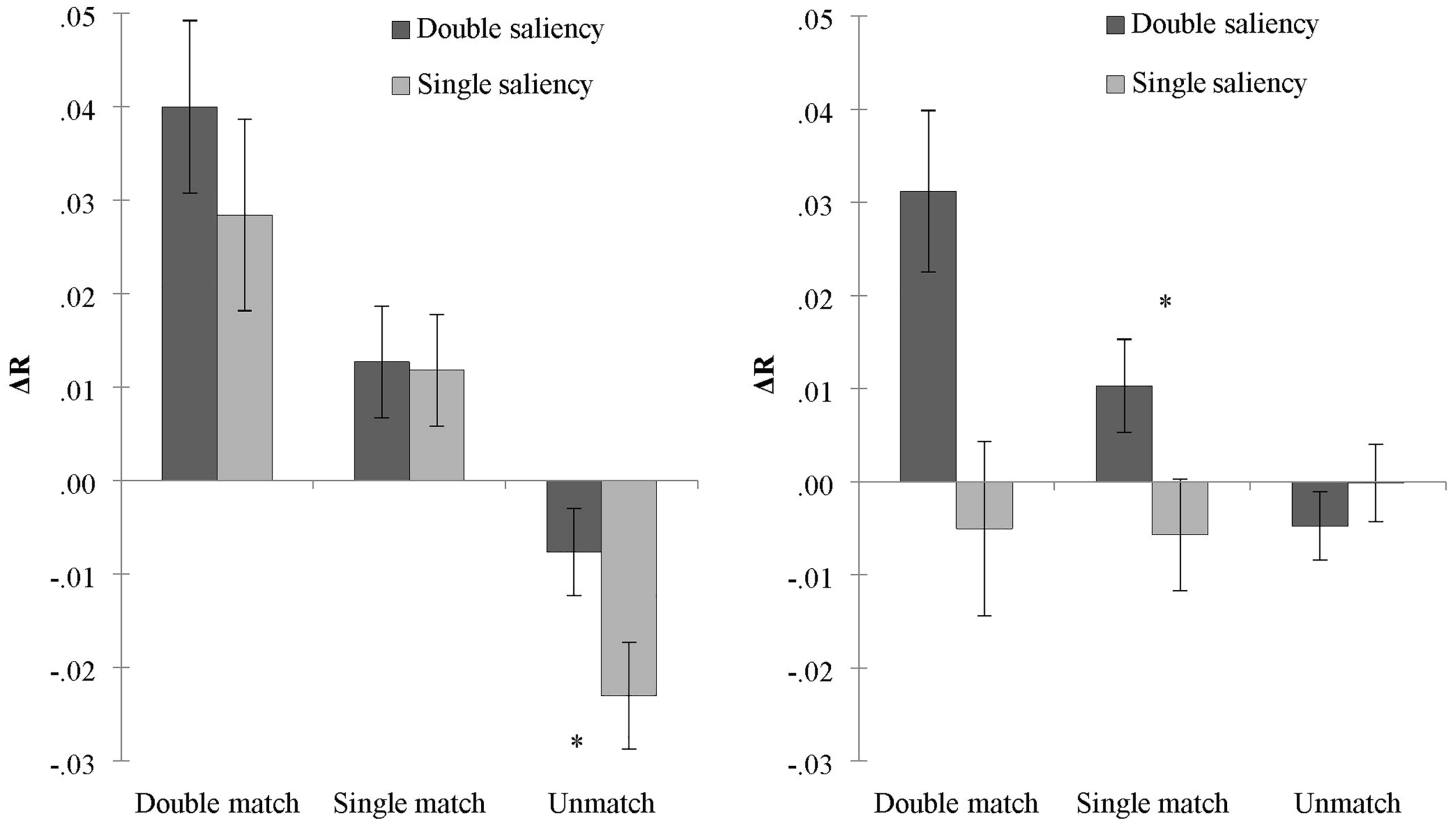
The RT differences of judgments under the conditions of single and double salience in Experiment 1 (left) and Experiment 2 (right). ^∗^*p* < 0.05.

To reveal the contribution of reward-prioritization to the difference in the processing of associations under each salience condition, we kept self-relevance in the same direction and calculated the difference of two associated shapes in each salience condition. Under the condition of double salience, *Δ*R_double_ = (RT_stranger-low reward_ − RT_self-high reward_)/(RT_stranger-low reward_ + RT_self-high reward_). Under the condition of single salience, *Δ*R_single_ = (RT _stranger-high reward_ − RT_self-low reward_)/(RT_stranger-high reward_ + RT_self-low reward_). To reveal the contribution of reward in two different salience conditions, we conducted a rANOVA of 2 (salience: double salience/single salience) × 3 (matching conditions: double match/single match/non-match) on the *Δ*R. The results are shown in Figure 3 (left). It was found that the main effect of salience was not significant, *F* (1, 32) = 2.27, *p* = 0.142, which indicated that the contrasting reward salience did not play a role in the difference (*ΔR*) of shape processing. The main effect of the matching condition was significant, *F* (2, 64) = 22.40, *p* < 0.001, $n_{p}^{2}$ = 0.41. *Δ*R in the double matching condition was greater than in the single matching and the non-matching conditions (*p*s < 0.001). *Δ*R in the single matching condition was greater than in the non-matching condition (*p* < 0.001). There was no significant interaction, *F* (2, 64) = 1.75, *p* = 0.18.

*Δ*R _double_ is the result of the addition of self-prioritization and reward-prioritization, and *Δ*R _single_ is the result of the subtraction of self-prioritization and reward-prioritization. In Experiment 1, *Δ*R _single_ was greater than zero, indicating that self-prioritization was greater than reward-prioritization under the condition of single salience. The difference between *Δ*R _double_ and *Δ*R _single_ was not significant. It further indicated that reward-prioritization did not make a prominent contribution to the processing of association.

However, there is a possibility that the impact of reward-prioritization in the condition of single salience is related to the amount of self-prioritization measured in the condition of double salience. Both dimensions may contribute to the processing of shapes but with different influences, depending on the specific context. To further verify this argument, we investigated the correlation between the two *Δ*Rs. We found a significant positive correlation between *Δ*R _double_ and *Δ*R _single_ in the double matching and single matching conditions, *r*_1_ = 0.500, *p* < 0.01 and *r*_2_ = 0.467, *p* < 0.01, but not in the non-matching condition, *r* = 0.167, *p* > 0.05. The positive correlation in matching conditions indicates that the result obtained by adding reward-prioritization (double highlight condition) or subtracting reward-prioritization (single highlight condition) remains the same on self-prioritization. Thus, compared with reward-prioritization, self-prioritization has a greater effect.

### Discussion

Combining the results of accuracy and RT, we found that, only in matching conditions (double matching and single matching), the processing of self-high reward shapes was more accurate and faster than that of stranger-low reward shapes, and the processing of self-low reward shapes was more accurate and faster than that of stranger-high reward shapes. In the non-matching condition, the processing of self-high reward shapes was less accurate and slower than that of stranger-low reward shapes, and the processing of self-low reward shapes was less accurate and slower than for the stranger-high reward shapes. In addition, there was no difference in *Δ*R under the conditions of single and double salience, and there was a significant positive correlation in *Δ*R between the conditions of single and double salience. Therefore, the results suggest that participants are more likely to associate with representations of self-relevance than with representations of the reward, which contributes to evidence of self-bias.

## Experiment 2

### Method

#### Participants

Thirty-two participants were randomly selected [13 males and 19 females, 18–27 years old, with an average age of 20.66 (*SD* = 1.75 years)]. All the participants were college students with normal or corrected vision. They participated voluntarily and had not participated in Experiment 1. Appropriate compensation was given after completing the experiment. Each participant was given monetary compensation ($15) after completing the experiment. The experimental protocol was approved by the Ethics Committee of the local University (masked for review).

All materials and procedures were identical to Experiment 1, except the social label that was changed from “stranger” to “friend.”

### Results

**Figure 4:**
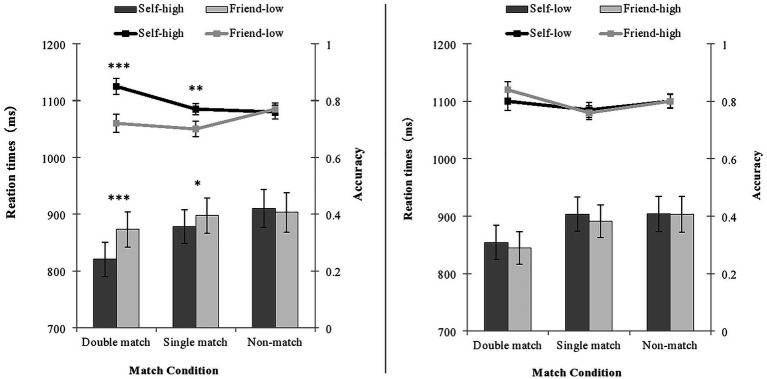
The accuracy and RT of judgments under the condition of double salience (left) and single salience (right) in Experiment 2. The line represents accuracy, and the bars represent the RT. ^∗∗∗^*p* < 0.001; ^∗∗^*p* < 0.01; ^∗^*p* < 0.05.

Outliers (RT less than 200 ms) were eliminated, accounting for 2.37% of the total data. A rANOVA of 2 (shapes: shape 1/shape 2) × 3 (matching condition: double match/single match/non-match) was conducted on the accuracy and RT of matching judgments. We analyzed conditions of double salience and single salience separately. The result is shown in Figure 4.

#### Double Salience

##### Accuracy

The results showed a significant main effect of shape, *F* (1, 31) = 21.83, *p* < 0.001, $n_{p}^{2}$ = 0.41. The matching judgment of self-high reward shapes was significantly more accurate than that of friend-low reward shapes. The main effect of the matching condition was not statistically significant, *F* (2, 62) = 2.22, *p* = 0.117. The interaction between the shape and the matching condition was significant, *F* (2, 62) = 14.32, *p* < 0.001, $n_{p}^{2}$ = 0.32. A paired sample *t*-test of shape was conducted on the three matching conditions. In the double matching condition and the single matching condition, the accuracy rate of processing self-high reward shapes was higher than that of friend-low reward shapes: *t* (31) = 5.37, *p* < 0.001, Cohen’s *d* = 0.96, for the double matching condition; *t* (31) = 3.00, *p* < 0.01, Cohen’s *d* = 0.61, for the single matching condition. There was no significant difference in the accuracy of processing the two shapes in the non-matching condition *t* (31) < 1.

##### Reaction Time

The main effect of the shape was significant, *F* (1, 31) = 7.54, *p* = 0.01, $n_{p}^{2}$ = 0.20. The RT on matching judgment of self-high reward shapes was significantly faster than that of friend-low reward shapes. The main effect of the matching condition was statistically significant, *F* (2, 62) = 19.61, *p* < 0.001, $n_{p}^{2}$ = 0.39. Participants responded faster in the double matching condition than they did in the single matching condition and the non-matching condition (*p*s < 0.001), with a faster RT in the single matching condition than in the non-matching condition (*p* = 0.011). The interaction between the shape and the matching condition was statistically significant, *F* (2, 62) = 17.15, *p* < 0.001, $n_{p}^{2}$ = 0.36. A paired sample *t*-test of shape was performed on the three matching conditions. In the double matching condition [*t* (31) = 3.99, *p* < 0.001, Cohen’s *d* = 0.71] and the single matching condition [*t* (31) = 2.32, *p* = 0.027, Cohen’s *d* = 0.41], the RT for processing self-high reward shapes was faster than that of friend-low reward shapes. In the non-matching condition, there was no significant difference in RT for processing the two shapes, *t* (31) = 1.00, *p* = 0.324.

#### Single Salience

##### Accuracy

There was no significant main effect of shape, *F* (1, 31) = 0.39, *p* = 0.537. The main effect of the matching condition was marginally significant, *F* (2, 62) = 3.03, *p* = 0.056, $n_{p}^{2}$ = 0.09. Participants responded more accurately in the double matching condition than they did in the single matching condition (*p* = 0.022). There was no significant difference between the matching and non-matching conditions (*p*s > 0.13). The interaction between the shape and the matching condition was not significant, *F* (2, 62) = 0.79, *p* = 0.457.

##### Reaction Time

The main effect of shape was not significant, *F* (1, 31) = 0.64, *p* = 0.43. The main effect of the matching condition was statistically significant, *F* (2, 62) = 13.18, *p* < 0.001, $n_{p}^{2}$ = 0.30. Participants responded faster in the double matching condition than they did in the single matching condition (*p* < 0.001) and the non-matching condition (*p* < 0.01). There was no difference between the single and non-matching conditions (*p* = 0.385). The interaction was not significant either, *F* (2, 62) = 0.46, *p* = 0.633.

#### Differences in Reward-Bias in Conditions of Double and Single Salience

We used the same calculation method from Experiment 1. Under the condition of double salience, we calculated the *Δ*R_double_ = (RT_friend-low reward_ − RT_self-high reward_)/(RT_friend -low reward_ + RT_self-high reward_). Under the condition of single salience, we calculated *Δ*R_single_ = (RT_friend-high reward_ − RT _self-low reward_)/(RT_friend-high reward_ + RT_self-low reward_). We conducted an ANOVA of 2 (salience: double salience/single salience) × 3 (matching conditions: double match/single match/non-match) on the *Δ*Rs. The results are shown in Figure 3 (right). The main effect of salience was significant, *F* (1, 31) = 7.98, *p* < 0.01, $n_{p}^{2}$ = 0.21. *Δ*R in the condition of double salience was greater than in the condition of single salience, which indicates that the different-directional reward played a role in the shape processing differences. *Δ*R was the combined effect of self-prioritization and reward-prioritization. Moreover, the main effect of matching conditions was also significant, *F* (2, 62) = 4.08, *p* = 0.022, $n_{p}^{2}$ = 0.12. *Δ*R in the double matching condition was greater than in the single matching condition (*p* = 0.016) and the non-matching (*p* = 0.046) condition. There was no difference between the single matching and non-matching conditions (*p* = 0.288). There was a significant interaction between salience and matching conditions, *F* (2, 62) = 13.03, *p* < 0.001, $n_{p}^{2}$ = 0.30. A paired sample *t*-test indicated that *Δ*R in double salience was greater than in single salience under the conditions of double matching [*t* (31) = 3.98, *p* < 0.001, Cohen’s *d* = 0.70] and single matching [*t* (31) = 2.45, *p* = 0.020, Cohen’s *d* = 0.44]. No difference was found in the non-matching condition (*p* > 0.05). In contrast to Experiment 1, which indicated self-prioritization as the main motivator, Experiment 2 indicated that the processing difference between the two shapes was a combined effect of self-prioritization and reward-prioritization.

Using a similar approach to Experiment 1, we conducted a correlation between *Δ*R_double_ and *Δ*R_single_ in Experiment 2. There was a significant positive correlation between *Δ*R_double_ and *Δ*R_single_ in double matching and single matching conditions, *r*_1_ = 0.499, *p* < 0.01, *r*_2_ = 0.411, *p* < 0.01, but not in the non-matching condition, *r* = 0.097, *p* > 0.05. The results suggest that compared to reward-prioritization, self-prioritization has a greater effect. Moreover, we compared the correlation coefficients in the two experiments and found that, under the two matching conditions, the correlation coefficients of the two experiments were not significantly different (*z*s < 0.27, *p*s > 0.79; Diedenhofen and Musch, 2015).

### Discussion

Experiment 2 partially replicated the findings of Experiment 1 by using a different social label of a friend instead of a stranger. In the non-matching condition, the processing of self-high reward shapes was not significantly different from stranger-low reward shapes, and the processing of self-low reward shapes was not significantly different from friend-high reward shapes. In both matching conditions, the processing of self-high reward shapes was quicker and more accurate than that of friend-low reward shapes. In the condition of single salience, participants responded more quickly and accurately to the double matching condition than they did to the single matching condition. In addition, *Δ*R in double salience was greater than the *Δ*R in single salience, which suggested that reward-prioritization contributed to the difference in shape processing. Therefore, the processing difference between the two shapes was the combined effect of self-prioritization and reward-prioritization when the social label of a friend was replaced with a stranger. However, we calculated *Δ*R by keeping self-bias in the same direction, so in terms of the positive correlation in *Δ*R between the conditions of double salience and single salience, reward-prioritization contributed less than self-prioritization.

## General Discussion

This study adopts the associative matching paradigm to investigate the effect of self-relevance and monetary rewards on perception. Previous studies have found that either self-prioritization or reward-prioritization plays an important role in human attention, memory, and perception (e.g., Anderson et al., 2011; Yin et al., 2019). Since Northoff and Hayes (2011) proposed the possible theoretical relationship between self and reward, very little research has been conducted to investigate how self-relevance and rewards simultaneously influence cognitive processes. Through two experiments, we explored the combined influence of self-relevance and monetary rewards in associative learning. In the experiments, participants were asked to learn shapes with two types of labels, namely, a social label (self vs. others) and a monetary reward (high vs. low). The results indicated that the participants responded more accurately and had a shorter RT in conditions of double salience (self-high vs. stranger or friend-low). However, accuracy and RT in conditions of single salience (self-low reward and other-high reward) were influenced by the social label (stranger in Experiment 1 and friend in Experiment 2).

Previous research study has shown that self-prioritization produces self-bias, and reward-prioritization produces high-reward bias (Sui et al., 2015, 2016). The central question in the present research study was how self-relevance and monetary reward influence cognitive processes simultaneously. Under the condition of double salience, the processing of self-high reward shapes (accuracy and RT in both experiments) was better than other-low reward shapes. However, the design could not disentangle the differences between self-prioritization and reward-prioritization when both factors simultaneously influenced the perception process in the same direction. Therefore, we created the condition of single salience, requiring participants to learn two single salience shapes: One shape represented self-low reward, which was also linked to self-prioritization, and the other shape represented other-high reward, which also represented reward-prioritization. The processing difference between self-low reward shapes and other-high reward shapes was based on the superposition in self-prioritization and reward-prioritization, which revealed the direction and size of the prioritization.

Specifically, we found that participants in Experiment 1 judged self-low reward shapes faster and more accurately than stranger-high reward shapes in both double and single matching conditions. This result indicated a prevalence of self-bias, i.e., that the role of self-prioritization is greater than reward-prioritization. Moreover, in the non-matching condition, the participants judged the self-low reward shapes slower and less accurate than stranger-high reward shapes. This result was similar to previous findings, indicating that stranger-related shapes yielded faster responses than self-related shapes in non-matching trials (Enock et al., 2018). The reason for this result may be that participants had more difficulties in making a mismatched “no” judgment for the self-low reward shapes, thus confirming a prevalence of self-related bias, but further evidence is still needed to confirm it. In sum, the reward-prioritization created by the high-low reward in Experiment 1 did not weaken the self-prioritization generated by self-relevance. However, there was no significant difference in Experiment 2 between the processing of self-low reward shapes and friend-high reward shapes in both matching and non-matching conditions. This result indicated that self-prioritization and reward-prioritization had similar effects in this context. In other words, there was no difference between the reward-prioritization produced by high-low reward and the self-prioritization produced by self-friend in Experiment 2.

The above results suggest that either self- or reward-prioritization plays a more important role in perceptual processing. However, the results cannot reveal the reward-prioritization size because self and reward directions were inconsistent. To reveal the contribution of reward-prioritization, we compared the difference of association processing (*Δ*R) under conditions of double and single salience. In Experiment 1, we found that differences between *Δ*R_double_ (self-high reward and stranger-low reward) and *Δ*R_single_ (self-low reward and stranger-high reward) were not significant, which indicated that the contrasting reward did not play a role in the difference (*Δ*R) of processing. In Experiment 2, we modified the social label from “stranger” to “friend.” We found that the *Δ*R_double_ (self-high reward and friend-low reward) was significant, while the *Δ*R_single_ (self-low reward and friend-high reward) was not significant. More importantly, the *Δ*R_double_ was greater than the *Δ*R_single_, which indicated that the same and opposite prioritizations derived by social labels and reward labels both contributed to the processing difference.

It is interesting to note that the direction of *Δ*R _double_ is always positive due to the high personal and reward salience of self-high reward shapes; its size reflects the combination of self-bias and reward-bias. However, how much each salience condition contributes to *Δ*R is impossible to calculate in this case. In addition, we tried to compare the *Δ*R _double_ in Experiment 1 and Experiment 2, and we found there was no significance between the two experiments, which might reflect the distance between self and the other (stranger or friend) being not relevant to the overall effect. Furthermore, the direction of *Δ*R _single_ is uncertain. If *Δ*R _single_ is positive, it would indicate that the contribution of self-relevance is more than that of reward salience. If *Δ*R _single_ is negative, it would indicate that the contribution of reward salience is more than that of self-relevance. For example, in the single salience condition in Experiment 1, the matching accuracy of self-low shapes was higher than stranger-high shapes, the RT for matching self-low shapes was shorter than stranger-high shapes, and *Δ*R _single_ was positive, which reflects a stronger effect of self-relevance than reward salience. However, in Experiment 2, there was no difference in performance between self-low shapes and friend-high shapes, and *Δ*R _single_ was not significant from zero, which likely reflects an equal contribution of self-relevance and reward relevance. Finally, in Experiment 1, we found negative effects in non-matching conditions, both in conditions of double salience and single salience. In other words, participants judged self-low reward shapes slower and less accurate than stranger-high reward shapes in the non-matching condition, possibly further supporting a prevalence of self-related bias. However, those results are not fully supported by the previous findings in the literature, so further evidence is needed to confirm this reasoning.

Comparing the results of Experiment 1 and Experiment 2, we found that, in associative learning, the role of reward-prioritization was greater when the social labels were self and friend. When the social label was a friend in Experiment 2, reward-prioritization contributed more to the difference in shape processing than when the social label was a stranger in Experiment 1.

One possible reason for this difference in shape processing is that the processing of self-related information and rewards uses the same value system. Individuals may assign different values to self-related information and high rewards, and subjective interpersonal distance may affect the value distribution of self-related information and high rewards. For example, in Experiment 1, the stranger had a small personal distance from the individual. This distance made the individual more sensitive to reward, so they assigned a high value to high-reward stimuli and a relatively low value to self-related stimuli, which led to a decrease in processing difference between self-related stimuli and high-reward stimuli (Sui and Humphreys, 2015d). In Experiment 2, the friend had a closer interpersonal distance to the individual. In this situation, individuals might have also been more sensitive to reward, as they assigned a high value to friend-high shapes associated with a relatively low value to self-low shapes. As a result, there was no difference in matching performance between self-low and friend-high shapes.

Another possible reason for the difference in shape processing is related to social comparison in the context of rewards. Close friends in the context of rewards may trigger a contrasting effect among individuals. When others are close friends, individuals are more sensitive to rewards (Wang et al., 2019). It is not a surprise that participants made better matching judgments in the condition of double salience (self-high reward vs. friend-low reward). However, in the condition of single salience (self-low reward vs. friend-high reward), there was no difference in matching judgment. When self and low reward were both common attributes of one association (friend and high reward were common attributes of another association), the participants recognized the contrasting effect of “friend-high reward and self-low reward.” This contrasting effect might have elicited negative emotions, which could have conflicted with positive attributes of the self (Ma and Han, 2010). This explanation is supported by previous studies that suggest threats of self-esteem and negative emotions can reduce or even cancel self-bias (Guan et al., 2012; Sui et al., 2016). Therefore, the roles of self-bias and reward-bias were not independent.

It should also be pointed out that participants in this study were not given corresponding reward feedback for reward-associated labels when rewarding the labels. Although reward learning can trigger implicit motivation, labels of the kind used here may not be enough to produce the expectations of rewards of the participants. Therefore, future studies should add external reward feedback to strengthen reward-related associations, such that the role of high and low rewards in both experiments may be revealed.

Since Northoff and Hayes (2011) proposed the three relationship models between self and reward, many studies have conducted similar studies on the relationship between self and reward and cognitive and neurological processing. We believe that there are substantial differences between self-bias and reward-bias in the functional sources of motivation. Self-bias comes from the internal self-improvement motivation of an individual, while reward-bias comes from the external reward motivation of an individual (Ryan and Deci, 2000). The two motivational aspects might work together, but their interaction may be affected by many factors. The current study integrated both self-bias and reward-bias in associative learning. The results demonstrated a general prevalence of self-bias over reward-related bias, with the latter significantly influencing the performance of the observer when the other is presented as a friend. Thus, a combined effect of self-bias and reward-bias was found. In future research studies, self-related information (self and others) and reward-related values (high and low) can be compared simultaneously with a neutral label as a reference condition in associative learning. When compared to the neutral label, the combined effect of self-bias and reward-bias can be further disentangled to see whether each dimension takes a different weight on perception.

In conclusion, the present research suggests a complex relationship between self-relevance and reward in perception as measured by an associative matching paradigm where the two forms of bias were presented simultaneously. When low self-relevance was represented by a stranger, the difference in associative learning was mainly an effect of self-bias. When low self-relevance was represented by a friend, the difference in associative learning was a combined effect of self-bias and reward-bias.

## Data Availability Statement

The raw data supporting the conclusions of this article will be made available by the authors, without undue reservation.

## Ethics Statement

The studies involving human participants were reviewed and approved by School of Psychology, Northeast Normal University http://psy.nenu.edu.cn/kxyj/llwyh.htm. The patients/participants provided their written informed consent to participate in this study.

## Author Contributions

LW contributed to the research conception, experiment design, data collection, and draft initial writing. YQ contributed to the data collection and draft initial writing. LH contributed to the experiment design, data collection, and draft initial writing. FJ contributed to draft the revised and final manuscript and supervise the revision. All authors contributed to the article and approved the submitted version.

### Conflict of Interest

The authors declare that the research was conducted in the absence of any commercial or financial relationships that could be construed as a potential conflict of interest.
